# Communications

**Published:** 1875-01

**Authors:** 


*Communications.*

Our sincere thanks are due for the following original communications, which we will publish soon as possible: "Rattlesnake Poison," etc., by Alban S. Payne, M.D., Markham, Virginia; "Dropsy of the Amnion," by J. C. Bishop, M.D., Middleport, Ohio; "Two Obstetrical Cases," by John Hockenhull, M.D., Cumming, Georgia; "Convulsions in Children—Cholera Infantum," by Wm. T. Beall, M.D., Edwards, Mississippi; "Lacerated Wound of the Perinæum," by E. T. Henry, M.D., Vicksburg, Mississippi; "Hydrocele in a Female," by G. A. Baxter, M.D., Chattanooga, Tennessee; "Growth and Nutrition Body," by Joshua B. Graves, Corning, New York.

